# Pediatric Migraine: Diagnosis and Management

**DOI:** 10.3390/jcm11247252

**Published:** 2022-12-07

**Authors:** Vincenzo Raieli, Vittorio Sciruicchio

**Affiliations:** 1 Child Neuropsychiatry Unit ISMEP—ARNAS Civico Palermo, 90134 Palermo, Italy; 2 Children Epilepsy and EEG Center, PO, San Paolo ASL, 70132 Bari, Italy

The WHO recognizes migraine as one of the most disabling diseases. Much has been clarified in terms of pathophysiology and diagnosis, and recently new horizons have opened up in treating migraine. However, this information, which comes mainly from studies conducted among adults, is also applied to children and adolescents. The available data, however, could be oversimplified with a risk of being misleading.

In fact, pediatric migraines are present starting from the first stage of development. Furthermore, they must be interpreted according to a dynamic evolutionary model that takes into account biological, psychological, and social aspects [1]. Preschool migraines must be differentiated, not only from migraine headaches in the adult population but also from migraines arising at school age and later in adolescence [1]. Therefore, the expectations regarding the treatment of child migraines are often different from those in treating adults [2].

There are numerous aspects regarding this condition requiring clarification, such as the chronobiological correlation between age and clinical aspects of migraine or the predictors of the clinical history of migraine in the short, medium, and long term. Furthermore, how lifestyle can influence headache features and what influences the response to drug therapy in children, which appears to be different from that in adults, should be investigated.

Further, little is known about what induces remission or resumption of migraine attacks throughout a subject’s life, thus necessitating both short- and long-term follow-up studies.

Overall, the scientific community’s interest in migraine in childhood has recently increased, as well as that in burden [3], physiopathological peculiarities [4,5], comorbidities [6,7], and lifestyle evaluation or modification [8,9].

At the developmental age, it is also easier to observe atypical clinical pictures due to clinical [10] and/or atypical topographical characteristics [11].

In recent years, a progressive increase in emergency department visits due to headaches has been registered, of which a conspicuous proportion are minors suffering from migraine attacks. Their management is difficult, and there is a need for both guidelines for therapy and avoidance of unnecessary testing [12,13].

The need to investigate the child population specifically derives not only from studies on migraine-afflicted adults (including human models), but also from pathogenetic, functional, and clinical-therapeutic studies from several areas of research suggesting that children are not simply “little adults” [14,15,16,17].

Currently, the most suitable model for interpreting a child’s migraine is a bio-psycho-social model that takes into account the genetic-neurobiological, psychological, and behavioral aspects corresponding to the child’s age and environmental conditioning [18]. Only from this perspective can the meaning of migraine for every child be grasped and, as result, an individualized diagnostic-therapeutic intervention can be planned. In our opinion, this is a very open field of research of interest for many researchers.

This Special Issue, “Pediatric Migraine: diagnosis and management”, aims to collect contributions from researchers focused on the specific peculiarities of migraine in young subjects, who are in a developmental period involving significant changes in their biology and psychology.

The main goal is to provide useful suggestions and open the field up for further reflections and research initiatives.
